# GABA_A_ Receptors Containing the α2 Subunit Are Critical for Direction-Selective Inhibition in the Retina

**DOI:** 10.1371/journal.pone.0035109

**Published:** 2012-04-10

**Authors:** Olivia Nicola Auferkorte, Tom Baden, Sanjeev Kumar Kaushalya, Nawal Zabouri, Uwe Rudolph, Silke Haverkamp, Thomas Euler

**Affiliations:** 1 Neuroanatomy, Max-Planck-Institute for Brain Research, Frankfurt/M., Germany; 2 Bernstein Center for Computational Neuroscience (BCCN), University of Tübingen, Tübingen, Germany; 3 Werner Reichardt Centre for Integrative Neuroscience (CIN), University of Tübingen, Tübingen, Germany; 4 Department of Biomedical Optics, Max-Planck-Institute for Medical Research, Heidelberg, Germany; 5 Laboratory of Genetic Neuropharmacology, McLean Hospital, Belmont, Massachusetts, United States of America; 6 Department of Psychiatry, Harvard Medical School, Boston, Massachusetts, United States of America; 7 Institute for Ophthalmic Research, University Hospital Tübingen, Tübingen, Germany; Dalhousie University, Canada

## Abstract

Far from being a simple sensor, the retina actively participates in processing visual signals. One of the best understood aspects of this processing is the detection of motion direction. Direction-selective (DS) retinal circuits include several subtypes of ganglion cells (GCs) and inhibitory interneurons, such as starburst amacrine cells (SACs). Recent studies demonstrated a surprising complexity in the arrangement of synapses in the DS circuit, i.e. between SACs and DS ganglion cells. Thus, to fully understand retinal DS mechanisms, detailed knowledge of all synaptic elements involved, particularly the nature and localization of neurotransmitter receptors, is needed. Since inhibition from SACs onto DSGCs is crucial for generating retinal direction selectivity, we investigate here the nature of the GABA receptors mediating this interaction. We found that in the inner plexiform layer (IPL) of mouse and rabbit retina, GABA_A_ receptor subunit α2 (GABA_A_R α2) aggregated in synaptic clusters along two bands overlapping the dendritic plexuses of both ON and OFF SACs. On distal dendrites of individually labeled SACs in rabbit, GABA_A_R α2 was aligned with the majority of varicosities, the cell's output structures, and found postsynaptically on DSGC dendrites, both in the ON and OFF portion of the IPL. In GABA_A_R α2 knock-out (KO) mice, light responses of retinal GCs recorded with two-photon calcium imaging revealed a significant impairment of DS responses compared to their wild-type littermates. We observed a dramatic drop in the proportion of cells exhibiting DS phenotype in both the ON and ON-OFF populations, which strongly supports our anatomical findings that α2-containing GABA_A_Rs are critical for mediating retinal DS inhibition. Our study reveals for the first time, to the best of our knowledge, the precise functional localization of a specific receptor subunit in the retinal DS circuit.

## Introduction

One of many neural computations performed by the retina is direction selectivity (DS), with certain types of retinal ganglion cells (RGCs) tuned to specific directions of image motion. Direction-selective ganglion cells (DSGCs) were first systematically studied in the rabbit several decades ago [1]. They robustly fire when presented with a light stimulus moving in a particular (“preferred") direction, but are silent when the stimulus moves in the opposite (“null") direction. Since then, three functionally distinct types of DSGCs have been identified: the originally described ON-OFF type in the rabbit [1], [2] makes up the most numerous population of DSGCs, has a bistratified morphology [3], [4] and detects brighter-than-background (“ON") as well as darker-than-background (“OFF") moving stimuli [5]. The other “classical" DSGC, the ON type [2], has monostratified dendrites [6], [7] and only responds to brighter-than-background stimuli. The ON and ON-OFF DSGCs can further be distinguished by their velocity tuning – the ON type prefers slower stimuli – and the fact that ON DSGCs detect global movement whereas ON-OFF DSGCs are specialized for local motion [5], [8]. Furthermore, ON and ON-OFF DSGCs comprise different functional subtypes [5], [6], [9], [10], [11], [12], [13]. Recently, in the mouse retina another GC was described that exhibits a DS phenotype: an OFF cell with a strongly asymmetrical wedge-shaped dendritic tree that is pointing ventrally [14]. It responds preferentially to motion from the soma to the dendritic tips, suggesting that the underlying DS mechanism makes use of the cell's highly asymmetric morphology. In contrast, most classical DSGCs have rather symmetrical dendritic arbors that do not necessarily correlate with the cell's preferred motion direction (but see [15]). These DGSCs rely mainly on spatially asymmetric inhibition from GABAergic interneurons, the starburst amacrine cells (SACs; for review see [16], [17], [18]), which generate local DS signals within their dendrites [19], [20], [21]. However, classical DSGCs may contain a subpopulation with more pronounced asymmetrical dendritic arbors, shown to contribute to DS responses, at least for lower motion velocities [15].

Thanks to the detailed knowledge of retinal organization and the possibility of easily testing even complex response paradigms in an intact neural network, retinal DS has long served as a popular model for neural computations (reviewed in [22]; see also [23], [24], [25]). It is now understood that the mechanism of DS relies on complex, genetically pre-programmed, highly selective spatial synaptic arrangements between its various elements (e.g. [26], [27], [28]). One important aspect in unraveling this complexity is identifying the specific neurotransmitter receptors involved and determining their precise localization in the circuit. This is particularly important for GABA receptors, since GABAergic connections play crucial roles at different levels in the DS circuitry. Pharmacological blockade of GABA_A_ receptors [29], [30], [31], [32] or ablation of the entire SAC population [33], [34] abolishes the directional tuning of classical DSGCs, while leaving their basic light responsiveness intact. Together with the co-fasciculation of SAC and DSGC dendrites within the inner plexiform layer (IPL), where the SACs form output synapses onto DSGCs [35], this indicates that the mechanism rendering DSGC responses directional rely on GABA_A_ receptor-mediated inhibition from SACs. Two studies investigated GABA_A_Rs in ON-OFF DSGCs [36], [37], showing antibody labeling against the α1 subunit in these cells. Here we identified GABA_A_ receptors containing the α2 subunit as the specific substrate of direction-selective inhibition from SACs onto DSGCs.

Using immunostaining and confocal microscopy, we show that GABA_A_R α2 clusters in the IPL of the rabbit and mouse retinae are concentrated in distinct strata, which correspond to the dendritic plexuses of SACs and DSGCs. We quantified the distribution of the GABA_A_R α2 subunit in both cell types, following labeling by dye injection in the rabbit, and show that these receptors are presynaptically aligned with SAC varicosities (the cell's output structures [35]) and postsynaptically with DSGC dendrites. By population recordings of light-driven GC activity [26] using two-photon calcium imaging [38] in GABA_A_R α2 knock-out mice [39], we functionally confirm that lack of the GABA_A_R α2 subunit leads to severe impairment in the DS tuning of RGCs. Taken together, our findings indicate that GABA_A_ α2-containing receptors are crucial mediators of directionally tuned inhibition in the retina.

## Results

### Candidate GABA receptor subunits in the DS circuit

**Figure 1:**
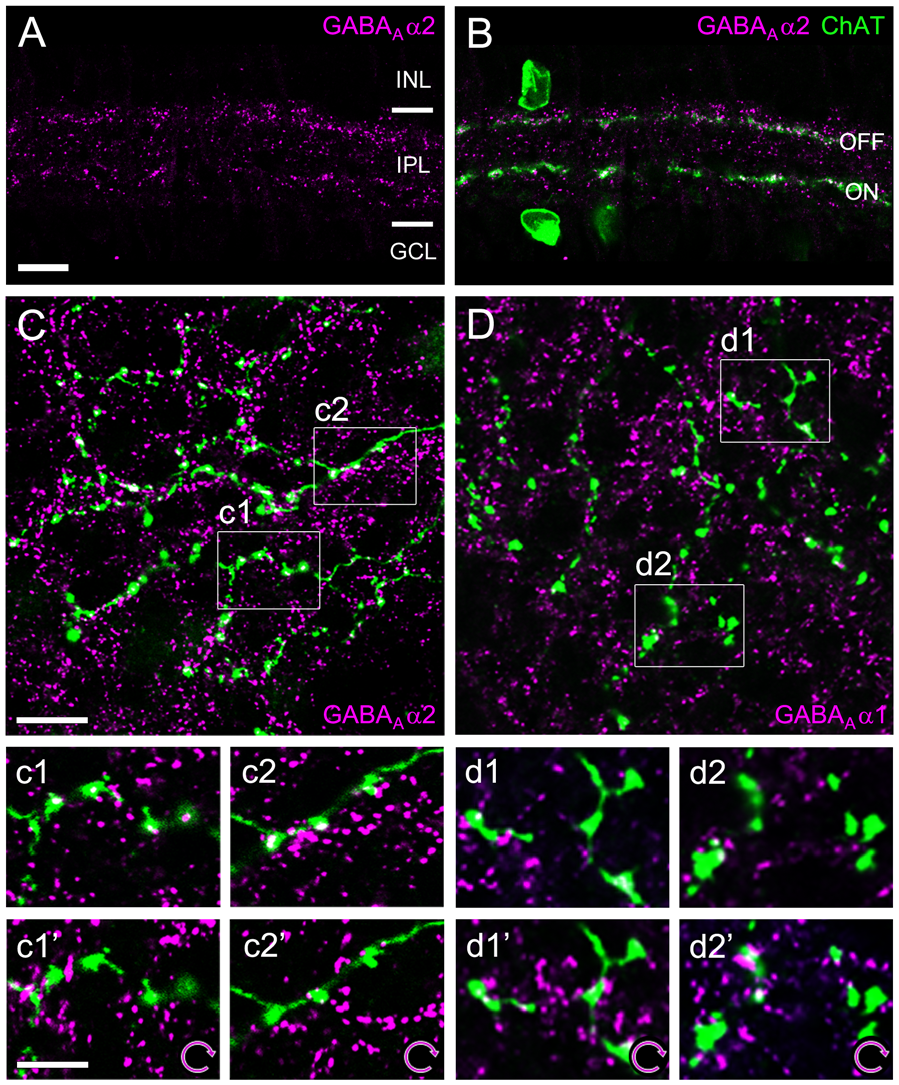
Localization of candidate GABA_A_R receptor subunits in relation to SAC dendrites and varicosities. **A–B**. Immunolabeling pattern of the GABA_A_R α2 subunit (*magenta*) in a vertical section of rabbit retina (single optical section; INL, inner nuclear layer; IPL, inner plexiform layer; GCL, ganglion cell layer). Starburst amacrine cells (SACs) were labeled against choline acetyltransferase (ChAT, *green*). ON SACs have somata in the GCL and their dendrites form the inner ChAT band; OFF SACs are located in the INL and their dendrites form the outer band. Synaptic receptor clusters (*magenta*) are unevenly distributed across the neuropile, with GABA_A_R α2 puncta concentrating in two bands along the SAC processes. **C**. Distal dendrites of a SAC injected with Neurobiotin (*green*) and co-stained with GABA_A_R α2 antibody (*magenta*) (single optical section): the majority of SAC varicosities are associated with receptor staining. Examples for such association are illustrated at higher magnification: c1 and c2 show observed receptor distribution, c1′ and c2′ show randomized control (magenta channel rotated 90° clockwise). Significantly fewer varicosities are associated with receptor clusters in the rotated control. **D**. Distal dendrites (*green*) of a SAC injected as in C but co-stained with GABA_A_R α1 (*magenta*) (single optical section): Some varicosities are associated with receptor clusters (see also magnifications in d1–d2). No obvious change in the degree of signal overlap is seen for the randomized control (d1′ and d2′). Scale bars: A, 10 µm (applies also to B); C, 10 µm (applies also to D); c1, 5 µm (applies to all insets).

Immunocytochemical studies of the retina have previously identified several different GABA_A_ receptor subunits in the IPL [40], [41]. Similar to what has been found for other receptor types (e.g. [42], [43]), synaptic clusters of GABA_A_Rs are not evenly distributed across the IPL. Of all GABA_A_ receptor subunits reported in the retina, only α2 and δ aggregate within the two IPL bands that can be labeled with antibodies against choline acetyl transferase (ChAT). These so-called cholinergic bands correspond to the dendritic plexuses of SACs, which in addition to GABA also use acetylcholine as a neurotransmitter [44], [45], [46]. It has been previously shown that the delta subunit of the GABA_A_R is exclusively expressed in SACs, where immunoreactivity was found in the soma and throughout the dendritic arbor [40] (data not shown). In contrast, GABA_A_R α2 immunoreactivity was found to be restricted to synaptic clusters in the IPL, which have also been attributed to the SACs [40]. However, since SAC processes tightly co-fasciculate with dendrites of DSGCs [35], [47], it is not clear whether the receptor staining is confined to SAC dendrites, DSGC dendrites, or present on both. Here we aimed at identifying the GABA_A_R that mediates directionally tuned inhibition from SACs onto DSGCs. Due to its selective enrichment in the cholinergic bands and its potential postsynaptic localization, GABA_A_R α2 stands out as a prime candidate (Fig. 1A–B, see also Fig. S4A–B). For comparison, we also examined the expression of GABA_A_R α1, the only GABA_A_R subunit reported in DSGCs so far [36], [37]. In the following, we analyzed the distribution of these two subunits on the presynaptic (SACs) and postsynaptic (DSGCs) circuit elements.

### Localization of GABA_A_R α1 and α2 at DS elements: presynaptic side (SACs)

Like many amacrine cells, SACs lack dedicated axons and instead use their dendrites for both synaptic input and output. SACs have radially symmetric dendritic arbors (Fig. S1), with 5–6 primary dendrites that ramify mostly in the distal third, where they show morphological specializations known as “varicosities" (e.g. [48]). Individual SAC dendrites receive synaptic inputs along their whole length, but their synaptic outputs are restricted to the distal third, the varicose zone [49]. Here, the presynaptic starburst element “wraps" around the postsynaptic DSGC dendrite, resulting in a characteristic hook-like formation (Fig. S1B–C) [35], [47].

Individually Neurobiotin-injected ON SACs in rabbit were co-stained with GABA_A_R α2 or GABA_A_R α1 and analyzed for immunolabeled receptor puncta tightly associated with varicosities (Fig. 1C–D), quantified as percentage of varicosities with puncta. Most varicosities (90%) contained at least one GABA_A_R α2 punctum and many (41%) contained multiple puncta (Fig. 1C and 3A). GABA_A_R α2 receptors could also be found on proximal SAC dendrites (n=2 cells, data not shown), but only at random incidence (p>0.05, 1-way ANOVA with Tuckey's multiple comparison test, see also *Methods*). GABA_A_R α1-containing receptors were more sparsely distributed: less than half of the varicosities (38%) were associated with single GABA_A_R α1 puncta and only 8% contained multiple puncta (Fig. 1D and 3A). In comparison, GABA_A_R α2 receptors were significantly enriched in the varicose zone of the SACs (p<0.001). To determine the chance level for co-localization of GABA_A_R puncta and SAC dendrites, we randomized the data by rotating one fluorescence channel of the micrographs by 90° and re-analyzed the distribution (Fig. 1c′–d′). We found only GABA_A_R α2 immunolabeling associated with varicosities at levels significantly higher than chance, while for GABA_A_R α1 we found no significant association (Fig. 3A–B).

### Localization of GABA_A_R α1 and α2 at DS elements: postsynaptic side (DSGCs)

**Figure 2:**
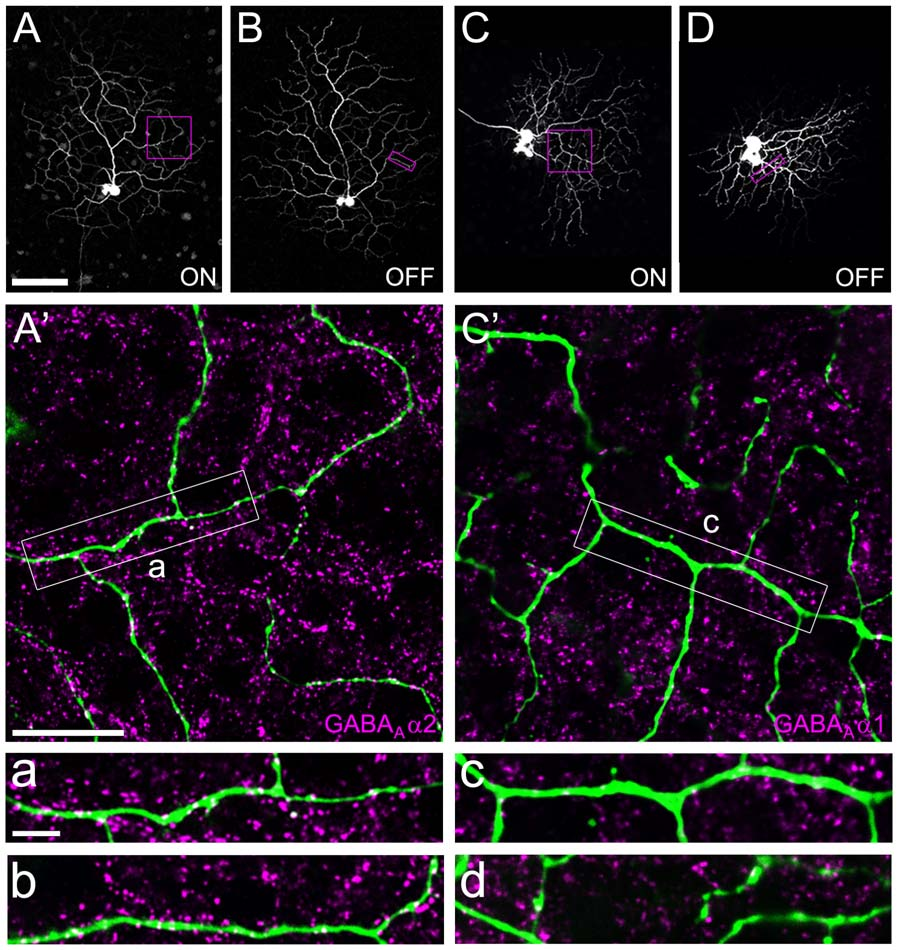
Localization of candidate GABA_A_R receptor subunits in relation to DSGC dendrites. **A–D**. Collapsed confocal stacks of two morphologically identified Neurobiotin-injected DSGCs (ON and OFF arbors shown separately). **A′**. Magnification of dendrite (ON layer) of the cell shown in A–B (*green*), co-stained with antibodies against GABA_A_R α2 (*magenta*). Dendrites are covered with receptor puncta, as evident at higher magnification in examples from the ON (a) and the OFF layer (b). **C′**. Magnification of dendrite (ON layer) of the cell in C–D (*green*), co-stained with GABA_A_R α1 antibodies (*magenta*). Only occasional receptor staining is found along the dendrites, as shown at higher magnification in examples from the ON (c) and the OFF arbor (d). Scale bars: A, 100 µm (applies also to B–D); A′, 20 µm (applies also to C′); a, 5 µm (applies also to b–d).

**Figure 3:**
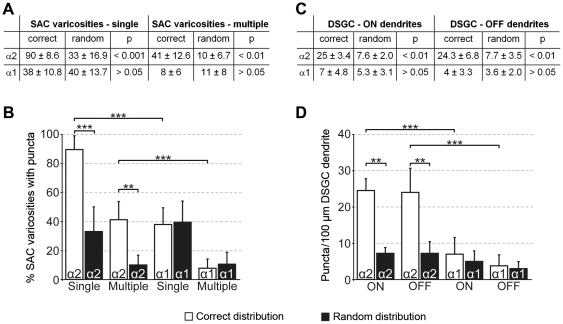
Quantification of candidate GABA_A_ receptor distribution on SACs and DSGCs. **A**. GABA_A_R α2 and α1 puncta distribution in relation to SAC dendrites in percentages of varicosities associated with at least a single punctum (=receptor cluster) and with multiple puncta (α2, n=245 varicosities from 5 cells in 4 retinae; α1, n=181 varicosities from 5 cells in 3 retinae). Percentages and standard deviation values are given for the original data (correct distribution) and for data with one fluorescence channel rotated by 90° (random distribution). **B**. Percentages of varicosities associated with single or multiple puncta plotted for the correct distribution (*open bars*) and randomized controls (*filled bars*). **C**. GABA_A_R α2 and α1 puncta distribution in relation to DSGC dendrites in number of receptor clusters per 100 µm of dendritic length for both ON and OFF dendritic arbors (α2, n=3 cells from 3 retinae; α1, n=3 cells from 2 retinae). **D**. Number of receptor clusters in ON and OFF sublayers plotted for the original data (correct distribution, *open bars*) and the randomized controls (random distribution, *filled bars*). Average and standard deviation values plotted in this figure were determined across cells.

We next analyzed the distribution of GABA_A_R α2 and GABA_A_R α1 along the dendrites of ON-OFF DSGCs, individually labeled by Neurobiotin injection (Fig. 2). Other than in the case of SACs, for which we were able to focus our analysis of receptor co-localization to synaptic structures (varicosities), the DSGC side displays no morphological specializations that can be recognized in light microscopy and reliably mark synaptic input sites. Therefore, we analyzed all co-localizations along dendrites and quantified receptor distribution as puncta per 100 µm of dendritic length. The dendritic arbor of the ON-OFF DSGC type occupies two distinct strata in the IPL: the ON dendrites co-stratify in the inner part with ON SACs, whereas the OFF dendrites co-stratify with OFF SACs in the outer IPL. We found similar distributions of both receptor subunits in the ON and the OFF layer. The α2 subunit was strongly co-localized with the ON and OFF dendrites, at densities of ∼25 puncta/100 µm dendritic length (Fig. 2a–b and 3C–D). The α1 subunit was also occasionally found on both DSGC arbors, however at much lower incidences: ∼7 and ∼4 puncta/100 µm dendritic length on ON and OFF arbors, respectively (Fig. 2c–d and 3C–D). Similar to SAC varicosities, the density of GABA_A_R α2 puncta on DSGC dendrites in both layers was significantly higher than that of GABA_A_R α1 (p<0.001, Fig. 3C–D). When compared to the respective randomized controls, only GABA_A_R α2 receptor density was found significantly higher than chance (Fig. 3C–D).

To determine the precise position of GABA_A_R α2 subunits at the SAC-DSGC synapse, we also studied the localization of this subunit by pre-embedding immuno-electron microscopy. While the tissue preservation was compromised due to the short fixation necessary for proper receptor staining, we were able to verify that the GABA_A_R α2 subunit clusters at membranes opposite to ChAT-immunolabeled SACs dendrites, at presumed SAC-DSGC synapses (Fig. S2).

Two previous studies described the distribution of GABA_A_R α1 receptors on ON-OFF DSGCs, addressing the possibility that the spatially asymmetric GABAergic inhibition impinging on ganglion cells is reflected by local (anisotropies with neighboring excitatory receptors, [36]) or global (overall receptor distribution across the dendritic arbor, [37]) asymmetries in the receptor distribution. Since our quantitative analysis shows a much higher density of GABA_A_R α2 puncta on ON-OFF DSGCs, the question arises whether previous attempts to find such asymmetries failed because they focused on the wrong receptor. We mapped the GABA_A_R α2-containing receptors of two anatomically identified ON-OFF DSGCs, one in mouse and one in rabbit retina (Fig. S3). While receptors can sometimes aggregate in larger clusters, they were generally evenly arranged along the dendrites. We did not find any obvious asymmetries in the receptor distribution, neither for the ON nor for the OFF dendritic arbor, in accordance with an early model of DS [46] and recent ultrastructural findings [47]. However, to exclude the presence of local anisotropies in the arrangement of inhibitory vs. excitatory receptors, more cells, preferably with identified preferred direction, need to be evaluated.

### Ganglion cell responses to moving bar stimuli in GABA_A_R α2 knock-out mice

**Figure 4:**
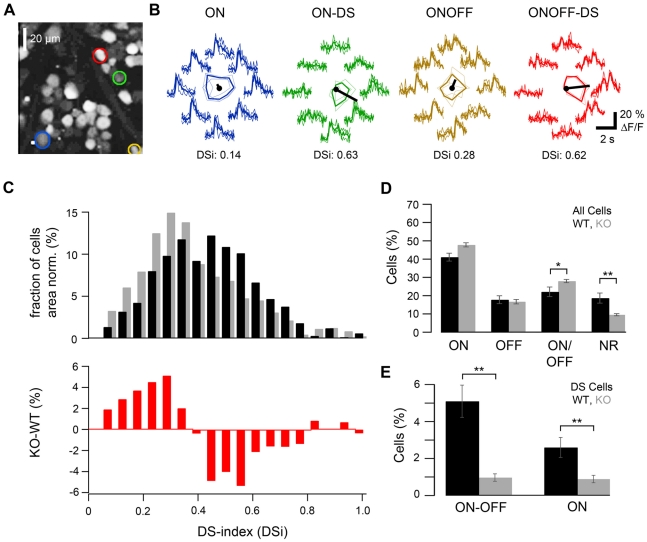
Analysis of DS responses in GABA_A_R α2 knock-out mice and wild-type animals. **A**. Two-photon micrograph showing an optical section (110×110 µm^2^, at the level of the GCL) of mouse retina, with 56 ganglion cells (and displaced amacrine cells) stained by the calcium indicator dye OGB-1 via electroporation. **B**. Calcium responses (ΔF/F) evoked by a bar stimulus moving in 8 directions measured in four exemplary GCs (trace color matches color of ROIs [regions of interest] in A): an ON (*blue*), an ON-DS (*green*), an ON-OFF (*yellow*) and an ON-OFF-DS (*red*) ganglion cell. Polar plots of the response amplitudes, with the preferred direction (*black line*) indicated, are shown in the center of the traces and reflect the different DS tuning strength of the cells (see also direction selectivity index, *DSi*; for definition see *Methods*). **C**. Histogram (*top*) showing the *DSi* distribution across all recorded GCL cells in GABA_A_R α2 knock-out mice (*gray bars*, n=2553 cells in 2 mice) and wild-type controls (*black bars*, n=1002 cells in 4 mice). *Bottom*: Difference between histograms (from top), illustrating the drop in the number of cells with higher DS-indices. **D**. Percentage of ON, OFF, ON-OFF, as well as non-responsive (NR) GCL cells in control (*black bars*) and knock-out animals (*gray bars*). **E**. ON-OFF and ON cells with a *DSi*>0.4 in the two groups of animals (cells were included or rejected after manual inspection of responses; see Results for complete criteria). (For E and D, relative cell type numbers were determined for each of the recorded GCL field –18 fields in wild-type, 30 in knock-out mice; with approx. 50–60 cells each– and then averaged; error bars indicate S.E.M.).

The conspicuous association of GABA_A_R α2 with SAC varicosities and DSGC dendrites suggests a critical role for this subunit in mediating inhibition in the DS circuit. To test this hypothesis, we measured ganglion cell calcium responses to moving light stimuli (Fig. 4) in knock-out (KO) mice lacking this specific subunit [39], [50]. GABA_A_R α2 KO mice appear healthy, with no obvious abnormal behavior [39]. Their retinae lack GABA_A_R α2 immunolabeling but otherwise show a completely normal gross anatomical organization (Fig. S4). Layer thickness and lamination, as well as SAC morphology and density were indistinguishable from the wild-type retina (data not shown).

To systematically screen for DS responses in the presence and absence of the GABA_A_ α2 subunit, we used two-photon population imaging of retinal tissue [38] that had been labeled with a synthetic calcium indicator via bulk electroporation [26] (Fig. 4A). We recorded the directional tuning of >3500 GCL cells (examples in Fig. 4B) in two KO (n=2553 cells in 30 fields, as the one shown in Fig. 4A) and four wild-type animals (n=1002 cells in 18 fields). We calculated the direction selectivity index (*DSi*) for every GCL cell that fulfilled a minimum response-amplitude criterion (see *Methods* for details). A small *DSi* indicates a more symmetrical spatio-temporal receptive field (Fig. 4B, *blue traces*), while a large *DSi* indicates a (presumed) DS cell (Fig. 4B: *green traces*, ON DS; *red traces*, ON-OFF DS). An unbiased comparison of the *DSi* distribution for all responsive GCL cells in the KO vs. wild-type mice revealed a dramatic reduction in the number of cells with higher *DSi*s (between 0.4 and 0.8; WT: 52%; KO: 33%), whereas the number of cells with low *DSi*s (<0.3) increased (Fig. 4C). Note that the percentage of cells for which the *DSi* changes may represent an overestimate of DS cells, because it may also include cells with asymmetric dendritic arbors (see *Discussion*). The change in *DSi* distribution is highly significant (p<0.01, using resampling and bootstrap percentile confidence intervals) and consistent with a critical role of α2-containing GABA_A_Rs in mediating DSCG directional tuning.

An advantage of such population recordings is that also potential “side-effects" of the receptor elimination on other retinal circuitries can be evaluated, for instance by comparing the relative frequency of the main functional types of GCs. We quantified the relative contribution of ON, OFF, ON-OFF and non-responding (NR) somata and averaged the data over the imaged fields (Fig. 4D). This revealed similar overall distributions of response polarities in wild-type and KO animals, suggesting that the lack of GABA_A_R α2 has no grave effects on other retinal circuits. Nevertheless, the KO retinae comprised a significantly higher fraction of ON-OFF cells and fewer NR cells compared to wild-type retina. This increase of light responsiveness in the KO is likely due to the overall reduction in GABA_A_R (α2) mediated inhibition and reflects unmasking of existing excitatory inputs. In support, similar results were obtained in the wild-type when applying GABA_A_ and/or GABA_C_ receptor antagonists (see below). For comparing the proportion of ON and ON-OFF DSGCs between genotypes, we considered all recorded cells that fulfilled the minimum response criterion, showed the correct response polarity (ON or ON-OFF), and had a *DSi*≥0.4. From this sample we also discarded, after visual inspection of their responses, cells with unstable baseline levels (e.g. change in baseline before light stimulation) or inconsistent responses over different trials of the same stimulus condition (e.g. cells that responded for less than 3 trials), but by definition also “non classical" types of DSGCs (see *Discussion*). Therefore, the percentage of DS cells resulting from this analysis (Fig. 4E) likely represents an underestimate. Nevertheless, also by this conservative but more subjective comparison, we found a highly significant drop in the number of both ON and ON-OFF DS cells in the GABA_A_R α2 KO retina (Fig. 4E).

We also analyzed our data with respect to the earlier described OFF DSGCs [14], which are not expected to be affected in the KO animals, since the dendrites of these cells stratify outside the IPL strata with pronounced GABA_A_R α2 density. However, we found only very few clear examples of OFF DSGCs in both the control (15 of 1002 cells) and KO mice (7 of 2553 cells, data not shown). It is possible that our stimulation parameters (i.e. positive contrast, motion velocity, stimulus size) were not optimal to detect these cells.

Analysis of the synaptic inputs to ON-OFF DS ganglion cells showed that not only inhibitory but also excitatory inputs are directionally tuned ([51], [52], [53], [54], but see [55]). Presynaptic GABAergic inhibition, i.e. at the terminals of bipolar cells, has been proposed as one of the potential underlying pathways. To test whether the remaining DS observed in the KO animals can be attributed to such a mechanism, we recorded RGC responses in the presence of GABA_A_ and GABA_C_ receptor blockers (50 µM gabazine, 50 µM TPMPA; data not shown). Blocking GABA Rs in the KO mouse strongly increased the overall light-responsiveness of the cells (mean response amplitude with GABAR block: 240% of control) and caused a shift in DSi distribution towards smaller values (mean *DSi* from Gaussian fit of histogram, control: 0.38±0.08, n=470 cells; GABA R block: 0.33±0.13, n=514 cells; p<0.05, T-Test; data not shown), indicating that additional GABAergic pathways that do not contain GABA_A_R α2 –possibly presynaptic inhibition at bipolar cell terminals– may contribute to the DS circuit. We still observed cells with high *DSi* values in the KO mice under this additional GABA R blockade (data not shown). This shows that GABA-independent response asymmetries (cf. [15]) contribute strongly to the DS as calculated by our analysis.

Taken together, our data strongly suggests that DS is severely impaired in the absence of GABA_A_R α2. This supports our anatomical findings that this receptor is located at the SAC-DSGC synapse (likely on the DSGC side), where it mediates direction-selective inhibition.

## Discussion

Many studies investigated the direction-selective circuit of the retina, as a model of a relatively simple neural computation. Our present understanding of the available data suggests highly complex synaptic arrangements that go beyond simple connectivity between cell types (see [47]), towards very selective local processing [19], [20], [21], [56]. It is therefore essential to gain knowledge about the underlying structural elements, particularly which neurotransmitter receptors are expressed at specific synapses on different cells in this circuitry. Here we provide the first compelling evidence for the precise localization of a specific receptor subunit in the DS circuit.

Two lines of evidence suggest that GABA_A_ receptors containing α2 subunits specifically mediate direction-selective inhibition from SACs onto DSGCs. First, our anatomical experiments show that α2 is not only strongly expressed within the cholinergic bands in the IPL ([40]; Fig. 1A–B, Fig. S4A–B), but is aggregated particularly at SAC varicosities (Fig. 1C–D, 3A–B) –the cell's synaptic output structures– and also at the postsynaptic side, significantly co-localized with DSGC dendrites (Fig. 2, 3C–D). This is in contrast to GABA_A_R α1 (Fig. 3), a subunit that has been previously reported to be present in ON-OFF DSGCs [36], [37]. Second, our population recordings in GABA_A_R α2 KO mice reveal a significant drop in directionally selective cells (Fig. 4C,E), whereas the overall light responsiveness and the relative frequencies of ON, OFF and ON-OFF retinal ganglion cells are largely the same as in the wild-type animals (Fig. 4D).

The majority of anatomical data was obtained in the rabbit, whereas for the recordings we used mice. Each model presents its advantages: rabbits are the classic animals for studies of direction-selectivity and facilitate selective targeting of DSGC somata, thanks to the very characteristic semilunar-shaped nuclei they exhibit in this species. On the other hand, mice are indispensable for the deeper understanding of DS circuitry function through the availability of genetically modified animals. Previous studies showed that the physiology of DSGCs in mouse and rabbit is very similar (e.g. mouse: [57], [58]; rabbit: [52], [53]), and our findings support the view that retinal direction-selectivity is based on analogous mechanisms in the two species.

### Receptor subunit localization at light-microscopical level

The 3D structure of the varicosities, obvious ultrastructurally [47], [49] and also apparent in our confocal micrographs (Fig. S1 B–C), makes it difficult to resolve the exact localization of associated receptors. Both the SACs' own receptors as well as those located postsynaptically, on the DSGC dendrites, will mostly appear co-localized with the varicosities in confocal microscopy. While we cannot exclude GABA_A_R α2 expression in SACs, we offer three lines of evidence for their postsynaptic localization: first, GABA_A_R α2 immunolabeling is significantly associated with SAC varicosities, the sites where SACs make output synapses, but not with the proximal dendrites, where mainly input is received (data not shown). While SACs might receive inputs also from other amacrine cells at the majority of varicosities, GABA_A_R α2 is also highly co-localized with DSGC dendrites, which we know from physiological studies to express GABA_A_ receptors, thus arguing for a postsynaptic localization. Second, our pre-embedding double labeling EM experiments indicate the presence of GABA_A_R α2 postsynaptically to SAC dendrites, on membranes most likely belonging to ganglion cells (Fig. S2). Third, should GABA_A_R α2 expression be restricted to SACs, one would not expect a clear phenotype in the GABA_A_R α2 KO mice, since GABA_A_R blockers do not interfere with SAC ability to generate DS signals [19], [20], [21], [58], and so direction selectivity downstream would remain intact.

### Remaining DS in GABA_A_R α2 knock-out mice

Direction selective cells were defined in our analysis of the physiological experiments as ON or ON-OFF cells with a minimum *DSi* of 0.4. This threshold was used in accordance with previous measurements of DS in the mouse retina [11], [12], [59], [60]. We found ∼5% of the cells in the GCL to be ON-OFF DS ganglion cells (and ∼2.5% ON DS), which is consistent with earlier reports using the same recording method [26], [47]. Since the population imaging does not reveal the morphological identity of the recorded cells, our analysis presumably also includes non-DS cells that reach a high *DSi* as a result of an asymmetrical dendritic arbor and/or active dendritic properties [15]. This is likely the main explanation for why we also found cells with high *DSi*s in the KO mice (Fig. 4C). Also the aforementioned OFF DSGCs, which have strongly asymmetrical dendrites and display light responses tuned to upward motion [14], likely contribute to the cells that remain DS in the KO animals, although we found only few convincing examples of such RGCs (data not shown). Asymmetric dendritic morphologies have also been described for several other GC types of the mouse retina (e.g.: B3, C6, D1 [61], some cells in clusters 6 [62], 2, 5, 6, 7, 8, 9 [63] or G_3_, G_5_, G_12_, G_18_ [64].) Furthermore, a recent study identified a subset of classical ON-OFF DSGCs, which are able to maintain directional tuning to slow moving stimuli even in the presence of GABAR blockers, due to active dendritic properties [15].

There is also a technical reason that may bias the responses to the moving bar stimulus such that also non-DS cells display some apparent directional tuning: The bar stimulus cannot be equally well centered on all cells in a recorded field, therefore the spatial offset between the cells location and the center of the stimulation field (i.e. where the trajectories of the moving bar stimuli cross) can in principle distort the resulting tuning curve. This effect would be augmented by asymmetric dendritic arbors – in line with recent observations [15]. Nevertheless, we did not see substantial differences in the distribution of directionally-tuned GCs across each recorded field (i.e. edge vs. center), likely because the recorded fields were relatively small (∼100×100 µm) compared to the dendritic tree diameters of mouse RGCs (∼100 to 250 µm, [64]). Since such a potential bias is resulting from the recording situation, it is expected to be the same for both KO and wild-type animals and therefore should not affect our conclusions about the response differences between the two genotypes. In addition, indirect network mechanisms, other than direct GABA transmission from SACs to DSGCs, may contribute to the remaining DS in the KO. Such mechanisms have been suggested to operate presynaptically through DS inhibition at bipolar cell terminals [51], [52], [53], [54], [65] or be mediated by asymmetric cholinergic excitatory inputs from SACs [56]. Our experiments in which GABA_A_ and GABA_C_ receptors were pharmacologically blocked in the GABA_A_R α2 KO (data not shown) support a role for presynaptic inhibition at bipolar cell terminals in retinal DS. The same experiments showed that cells with high *DSi* values persisted under these conditions, consistent with the finding that asymmetric dendritic morphology and/or active dendritic properties [15], [66] provide sufficient directional tuning in at least some DSGCs.

### Specific receptor subtypes for specific functional roles

GABA_A_ receptors are pentamers generally composed of two α, two β and one γ subunit and mediate fast synaptic and tonic extrasynaptic inhibition (for review see [67]). Much evidence has accumulated from immunocytochemical, physiological and pharmacological studies throughout the central nervous system supporting that specific receptor subtypes are targeted to distinct neural circuits [67], as well as to specific synaptic sites within one neuron [68], [69], [70], [71]. This pattern has been consistently demonstrated in the retina, where subunit receptor expression has been extensively studied for both excitatory and inhibitory receptors [43], [72], [73], [74], [75]. Here we report selective expression of GABA_A_R α2, but not α1, at a specific synapse in the DS circuit, supporting the idea that these subunits belong to different receptors and, likely, different synaptic complexes. Despite the fact that GABA_A_ α1 receptor density that we counted in ON-OFF DSGCs did not reach levels significantly higher than chance, we cannot exclude low expression of these receptors, possibly at synapses other than from SACs (outside the DS circuit). Interestingly, the choice for receptors in the DS circuit appears to be conserved between pathways (ON and ON-OFF; Fig. 4) and among species, as suggested by our findings in rabbit (Fig. 1) and mouse (Fig. S4, Fig. 4), as well as by those reported previously in rat [40]. As for the complete structure of the GABA_A_ receptor expressed postsynaptically in the DSGCs, judging from the composition of most GABA_A_Rs found in the higher brain, α2β3γ2 is the most likely candidate [67].

So far no prominent functional characteristics have been reported for GABA_A_R α2. Therefore, it is unclear whether and how this specific subunit could support DS computation other than “simply" relaying asymmetric inhibition from SACs. On the other hand, it is surprising to find a specific subunit almost exclusively at a very particular location in a neuronal circuit, especially when the circuit relies on highly complex arrangements at synaptic level – as retinal DS does. In view of the extensive rewiring that appears to take place in the DS circuit within 1–2 days during development (e.g. [27], [28]), it is possible that specific subunits at distinct locations may facilitate the differential targeting of receptors to different synapses. In this scenario, a particular subunit may play less a functional but more an organizational role.

Notably, the δ subunit, one of the less common GABA_A_R subunits, was previously shown to be expressed in the retina selectively by SACs [40] and the responsible *Gabard* gene was recently identified as one of several specific markers of SACs in a wide screening of adult retinal cell types' transcriptomes [S. Siegert, Friedrich Miescher Institute for Biomedical Research, personal communication]. In other brain regions, e.g. dentate gyrus and cerebellum, δ-containing GABA_A_ receptors mediate tonic inhibition and are activated by low ambient GABA levels [76]. It is tempting to speculate on possible interactions of δ and α2 subunits, should SACs be shown to express the latter as well. However, to our knowledge, the presence of the α2 and δ subunits in the same receptor complex *in vivo* has not been reported. More likely, δ and α2 belong to different GABA_A_Rs located in SACs and DSGCs, respectively (see discussion on receptor localization above). Nonetheless, they could be localized at the same synaptic site, for example with presynaptic GABA_A_R δ serving as modulators for the DS signals passing downstream.

## Materials and Methods

### Animals and tissue preparation

All animal procedures were carried out in accordance with institutional guidelines of the Max-Planck-Institute for Brain Research, Frankfurt, and the University of Tübingen, following the standards described by the German animal protection law (Tierschutzgesetz). Euthanasia of both mice and rabbits for organ harvesting (retinae) used in this study has been approved by the animal welfare officer of the respective facility (MPI for Brain Research and University of Tübingen) and reported to the local authorities (Regierungspräsidium Darmstadt and Regierungspräsidium Tübingen, respectively).

For single-cell dye injections, adult New Zealand White rabbits (Charles River Laboratories) were deeply anesthetized by intramuscular injection of ketamine (50 mg/kg body weight; Curamed Pharma/Pharmaselect) and xylazine (Rompun, 10 mg/kg body weight; Bayer) and then sacrificed with intravenous pentobarbital (Narcoren, 160 mg/kg body weight; Merial). The eyes were quickly enucleated, and the retinae were dissected in carboxygenated (5% CO_2_, 95% O_2_) Ames medium (Sigma-Aldrich, Munich, Germany). For physiological recordings, adult (>4 weeks old) wild-type and GABA_A_R α2 knock-out (mutant allele Gabra2^tm2.2Uru^ on C57BL/6J background) [39], [50] mice were dark adapted for at least one hour prior to experiments. All further tissue handling was carried out under dim red illumination. Mice were anaesthetized with Isoflurane (Baxter, Unterschleißheim, Germany) and killed by cervical dislocation. Eyes were enucleated, transferred to carboxygenated Ringer's solution (Biometra, Göttingen, Germany) and the retinae dissected into two halves. For LM immunocytochemistry, the posterior eyecups of adult wild-type and GABA_A_R α2 knock-out mice were fixed with 4% paraformaldehyde in 0.1M phosphate buffer (PB) for 10 minutes at room temperature (RT). For electron microscopy, eyecups were fixed in 4% paraformaldehyde, 0.05% glutaraldehyde and 0.2% picric acid in 0.1M PB for 25 minutes at RT.

### Cell injections and immunolabeling

A piece of flat mounted rabbit retina, with the ganglion cell layer on top, was placed under the microscope and perfused with warmed (32°C), carboxygenated medium. The tissue was visualized using custom built infrared scattered light detection system (designed by W. Denk, MPImF) on a custom built eyecup scope ([77]; for details see also section *Two-photon calcium imaging*). Cells in the ganglion cell layer were targeted for injection: displaced (ON) SACs were identified by their small round somata (10 µm in diameter); DSGCs were recognized by their medium-sized cell bodies (16 to 20 µm) with semi-lunar nuclei. These somata were impaled with microelectrodes (borosilicate glass with filament, O.D. 1.0 mm, I.D. 0.58 mm; 100–200 MΩ) filled with 4% neurobiotin in 3M KCl. A current of 0.5 to 1.0 nA was applied for 3 to 5 minutes using a multiclamp amplifier (Molecular Devices GmbH, Ismaning, Germany). Small concentrations of Alexa-488 were added to the pipette solution to visualize the electrode tip during injections and to check the morphology of the injected cells immediately after injections. The electrode was then retracted carefully and the tissue was left to rest for about 30 minutes. Afterwards, the retina was fixed with 4% paraformaldehyde in PB for 10–20 minutes at RT. After rinsing in PB (4×15 min), the tissue was preincubated for 2 h in blocking solution (10% normal donkey serum, 1% bovine serum albumin, and 1% Triton X-100 in PB) at RT. Neurobiotin was revealed with Streptavidin-Alexa Fluor 488 (Invitrogen, Darmstadt, Germany, Cat.No. S-11223) diluted 1∶1000 in 3% normal donkey serum, 1% bovine serum albumin, and 1% Triton X-100 in PB, and incubated overnight at RT or for 3 days at 4°C. Receptor antibodies were usually incubated together with the Streptavidin. Antisera that recognize specific amino acid sequences of GABA_A_ receptor subunits alpha 1 and 2 (GABA_A_R α1 and GABA_A_R α2) were used: GABA_A_R α1 was stained with a monoclonal antibody raised in mouse (Chemicon International, Temecula, CA, USA, Cat. No. MAB339), working dilution 1∶50; GABA_A_R α2 was labeled with a polyclonal rabbit antibody (Synaptic Systems, Göttingen, Germany, Cat. No. 224103), working dilution 1∶2000. Some retinae were also co-stained for choline acetyl transferase (ChAT), with a polyclonal antibody raised in goat (Chemicon, Cat. No. AB144P). Following incubation with the primary antibody–Streptavidin mix, the tissue was washed several times in PB, then incubated for 2 h at RT with the secondary antibody diluted 1∶500 in the same incubation solution as above. The following secondary antibodies were used: donkey-anti-mouse Cy3 (Jackson ImmunoResearch, West Grove, PA, USA, Cat. No. 715-165-150), donkey-anti-rabbit Cy3 (Jackson ImmunoResearch, Cat. No. 711-165-152) and donkey-anti-goat Cy5 (Jackson ImmunoResearch, Cat. No. 705-175-003). Retinae were finally washed in PB, mounted in Aqua Polymount medium (Polysciences, Eppelheim, Germany) and stored at 4°C until visualized by confocal microscopy.

For immunolabeling on cryostat sections, the fixed mouse retinae were cryoprotected in graded sucrose (10%, 20%, and 30%) and sectioned vertically (16 µm) with a cryostat. Sections were labeled for calbindin, with a polyclonal antibody raised in rabbit (Swant, Bellinzona, Switzerland, Cat. No. 300), working dilution 1∶2000, and for protein kinase C alpha, with a monoclonal mouse antibody (Biodesign, Saco, ME,USA, clone MC5), working dilution 1∶100.

### Pre-embedding immunoelectron microscopy

The localization of GABA_A_R α2 subunit was investigated at the ultrastructural level using a pre-embedding double labeling method in combination with ChAT [78]. A short fixation (25 min) in 4% paraformaldehyde, 0.05% glutaraldehyde and 0.2% picric acid was necessary to preserve the immunoreactivity adequately. After fixation, and cryoprotection, the tissue was frozen and thawed, and vertical vibratome sections (60 µm) were cut. Vibratome sections were blocked for 2 hours in PBS containing 10% NDS and 0.2% acetylated BSA (BSAc, Aurion, Wageningen, Netherlands), then incubated in anti-GABA_A_R α2 (rabbit, dilution 1∶500, Synaptic Systems) and anti-ChAT (goat, dilution 1∶100, Chemicon) with 3% NDS, 0.2% BSAc in PBS for 4 days at 4°C. Donkey anti-goat biotin-conjugated (Vector, Burlingame, CA) and donkey anti-rabbit conjugated to 0.8 nm nanogold particles (Aurion) secondary antibodies were applied (1∶100 and 1∶50, respectively), followed by an immunoperoxidase reaction (Vectastain Elite ABC kit; Vector) to visualize the biotinylated antibody. The sections were then post-fixed with 2.5% (v/v) glutaraldehyde in cacodylate buffer for 1 hour. The product of the enzymatic reaction and the nanogold particles were silver intensified using the silver enhancement system (R-Gent) from Aurion, as described by the manufacturer. The sections were then incubated in 0.5% (w/v) osmium tetroxide for 30 minutes at 4°C. Dehydration was carried out by an ethanol series and propylene oxide. Specimens were embedded in Epon, and a series of ultrathin sections (60 nm) were collected on copper grids and stained with uranyl acetate and lead citrate. Ultrathin sections were examined with a Leo 912 AB Omega transmission electron microscope (Carl Zeiss SMT AG, Oberkochen, Germany) and photographed with a wide-angle Dual Speed 2K-CCD camera in combination with ImageSP (TRS, Moorenweis, Germany) software.

### Confocal microscopy and image analysis

Image stacks were acquired with a confocal microscope (Olympus Fluoview FV1000) equipped with Helium-Neon and Argon lasers. Cell morphology was documented at low magnification, using a UPlanSApo 20×/0.75 objective. For quantitative analysis, high-resolution image stacks (1024×1024 pixels at 0.5 µm Z-intervals) of individual dendrites were obtained using a UPlanSApo 60×/1.35 oil objective. Image resolution was within the confidence range of the Z-axis resolution of the microscope (0.46 µm for the 60× oil objective). Micrographs used for illustration were cropped and adjusted for contrast and brightness in Photoshop 11.0 (Adobe Systems GmbH, Munich).

Synaptic puncta that were co-localized with SAC varicosities or DSGC dendrites were documented manually throughout the stacks. Receptor clusters were included in the analysis only when they showed at least 75% overlap with the dendritic profiles. In the case of DSGCs, the total length of dendrites within a collapsed image stack was measured and the number of co-localized synaptic puncta was normalized per 100 µm dendritic length to allow comparison among samples. To assess whether the density of receptors on varicosities and dendrites reached higher-than-chance levels, we generated random superimpositions of the receptor and cell labeling (by rotating one of the signals by 90°) and repeated counting. The value obtained was defined as chance level co-localization. Incidence levels of synaptic labeling were tested against the randomized controls by one-way analysis of variance (ANOVA) followed by Tuckey's multiple comparison test, using Prism 5 software (GraphPad Software, Inc., La Jolla, CA, USA).

### Bulk electroporation and two-photon calcium imaging

Bulk electroporation of fluorescent calcium indicator dye into the ganglion cells was carried out as described previously [26]. In brief, each retina half was mounted ganglion cell side up on a filter membrane (Anodisc 13, 0.2 µm pore size, Whatman, Maidstone, UK) and positioned between two 4 mm horizontal plate electrodes (CUY700P4E/L, Nepagene/Xceltis, Meckesheim, Germany). A 10 µl drop of 5 mM Oregon Green-BAPTA-1 hexapotassium salt (Invitrogen, Darmstadt, Germany) in Ringer was suspended from the upper electrode and lowered onto the specimen. After the application of 10 pulses (+11 V, 10 ms pulse width, at 1 Hz) from a pulse generator/wide-band amplifier combination (TGP110 and WA301, Thurlby Thandar/Farnell, Oberhaching, Germany), the tissue was moved to the recording chamber of the microscope (see below), where it was continuously perfused with carboxygenated Ringer at ∼37°C and left to recover for ∼60 minutes before the recordings started.

We used a custom built two-photon microscope [77] equipped with through-the-objective light stimulation and two detection channels for fluorescence imaging (red, HQ 610 BP 75, and green, D 535 BP 75; Chroma, Tübingen, Germany). A mode-locked Ti/sapphire laser (MaiTai, Spectra Physics, Newport, Santa Clara, CA) tuned to 927 nm served as the excitation source. To visualize the retinal structure in the red fluorescence channel, ∼0.1 µM of sulforhodamine 101 (Sigma-Aldrich, Munich, Germany) was added to the bathing medium. Changes in fluorescence reflecting Ca^2+^ activity were recorded from the ganglion cell somata in the green channel. A light stimulator [77] based on a miniature liquid-crystal-on-silicon (LCoS) display (i-glasses; EST, Kaiserslautern, Germany) and driven by custom-written software was used to project moving bar stimuli onto the retina. The stimulus consisted of a bright bar (300×1000 µm, at 3.87•10^3^ R*/s/cone) on a dark background (0.28•10^3^ R*/s/cone) moving in eight angular directions at 0.5 mm/s (n=3 trials per direction). Although color was supported, we restricted the stimuli used here to “gray" stimuli (with both blue and green stimulator components at the same opsin photoisomerization rate). Typically, 110×110 µm fields comprising 50–60 GCL cells at a time (for example see Fig. 4A) were recorded at 64×64 pixels (at 2 ms per line=7.8 Hz frame rate) using custom-written software (CfNT, M. Müller, MPImF, Heidelberg). A synchronization signal was acquired (@ 500 Hz) together with the image data to allow matching calcium signals with the stimuli.

### Analysis of Ca^2+^-imaging data

Images were analyzed offline using IgorPro 6.2 (Wavemetrics, Lake Oswego, OR, USA). Regions of interest (ROIs) were manually selected around each soma in a field of view and Ca^2+^ (ΔF/F_0_) signal traces were extracted using the freely available image processing package SARFIA [79]. Responses to individual stimulus presentations were extracted using the synchronization signal from the light stimulator, embedded in the image data. Note that we did not attempt to distinguish between RGCs and displaced amacrine cells, of which the latter make up almost 60% of the GCL cells in mouse retina [80]. It is conceivable that the fraction of non-responsive (NR) cells mainly consists of displaced amacrine cells; however, one cannot exclude also amacrine cells exhibiting light-evoked somatic Ca^2+^ changes (for discussion see also [26]). All further analysis was carried out using custom-written IgorPro routines. To quantify Ca^2+^ responses (e.g. Fig. 4B), the area (*A*) under the ΔF/F_0_ trace comprising both the ON and the OFF response (to the bar's leading and trailing edge, respectively), was calculated for each stimulus direction, resulting in 8 response vectors (*v*). To limit inclusion of cells with Ca^2+^ responses too close to noise, we discarded the 35%-fraction of cells with the smallest responses (“minimum response criterion"). For the remaining cells, a directional selectivity index (*DSi*) was determined as follows: 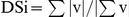. It ranges from 0 and 1, with small values indicating a more symmetrical spatio-temporal response profile and high values indicating directional tuning.

To test whether or not the difference in *DSi* distribution of knock-out vs. wild-type (Fig. 4C) is statistically significant the distributions were resampled (n=1000) and compared using the bootstrap percentile confidence interval (build-in IgorPro functions). To statistically compare the percentage of (manually classified) ON, OFF, ON-OFF and NR cells (Fig. 4D) as well as ON and ON-OFF DSGCs (Fig. 4E) in wild-type and knock-out we determined the respective mean percentage (and the standard error) by averaging across the recorded fields. Note that when plotting these percentages over the time-course of the experiments we found no detectable trend in the relative fractions for the different functional cell types, indicating that recording quality was constant (data not shown).

## Supporting Information

Figure S1 **Morphology of varicosities on the distal dendrites of starburst amacrine cells (SACs).** **A**. Distal dendrites of a dye-injected SAC (see inset) in a rabbit retina. **B–C**. High-magnification examples of varicosities, which are hook-like formations resulting from the presynaptic dendrite wrapping around the postsynaptic element [35], [47]. Scale bars: A, 15 µm (inset, 50 µm); B–C, 2 µm.
(TIF)

Figure S2 **Electron micrographs of ultrathin sections of mouse retina, pre-embedded and double labeled for ChAT and GABA_A_R α2.** Single section shown in **A**, two serial sections shown in **B′–B″**. The GABA_A_R α2 subunit is labeled with gold particles (*tiny black dots* indicated by *arrows*), which are visible on postsynaptic membranes apposed to ChAT-labeled SAC profiles, stained with DAB (*big black dots*, entire profiles overlaid in *green*). Note that the postsynaptic profiles do not contain vesicles, and are thus putative ganglion cell processes (compare to profile marked with *asterisk* in B′, which illustrates a typical presynaptic element, full of synaptic vesicles.) In B″ also note the elongated microtubule-like structures along the postsynaptic profile, typical for ganglion cell dendrites. Scale bar 0.5 µm.
(TIF)

Figure S3 **Spatial distribution of GABA_A_ α2 receptor staining across the dendritic arbors of direction-selective ganglion cells (DSGCs).** **A–B**. Reconstructions of putative ON-OFF DSGCs in mouse (A) and rabbit (B). The cell in the mouse retina was labeled in a transgenic GFP-O line, described in detail in [81]. ON and OFF dendritic trees are shown separately, with receptor puncta in *red* for the ON layer and *blue* for the OFF layer. In A, each dot represents a single receptor cluster (=an immunoreactive punctum); in B, dot size encodes number of receptor clusters (see scale beneath). Numbers indicate receptor cluster density per 100 µm dendritic length. The higher density of puncta found for the rabbit DSGC could reflect differences between species or, more likely, fixation degree. No obvious asymmetries can be seen across either cells or layers, as expected from recent statistical EM data [47] and illustrated in **C**. (While the values for the quadrants in B differ to some degree, regions with higher and lower densities are not segregated to particular sides of the dendritic field, e.g. null vs. preferred side.) C. SACs synapse onto DSGCs with different preferred directions (PD) with the constraint that each DSGC type is contacted by only those SAC dendrites oriented approximately along the DSGC's null direction (ND) (*left*). Together with the intrinsic DS tuning of the SAC output, this spatial arrangement warrants that SAC inhibition selectively vetoes the response to motion in null direction. Repeating this connectivity pattern for all SACs overlapping one given DSGC (*middle*) results in a relatively even distribution of receptors across the DSGC's dendritic tree (*right*). Scale bars: 100 µm.
(TIF)

Figure S4 **Retinal gross organization in GABA_A_R α2 knock-out mice.** Vertical sections of wild-type (A–D) and GABA_A_R α2 KO retinae (A′–D′) immunolabeled against GABA_A_R α2 (*magenta* in A–A′ and B–B′), choline acetyl transferase (ChAT; *green* in B–B′), calbindin (C–C′), and protein kinase C alpha (D–D′). The retinae of the KO animals are void of α2 receptor staining, but otherwise do not show any obvious difference in gross retinal organization (e.g. thickness or lamination) when compared to the wild-type. Scale bars: 20 µm (B′ applies also to A, A′, B; C′ applies to C; D′ applies to D).
(TIF)
