# Supplementary figures and images for: GABAA Receptors Containing the α2 Subunit Are Critical for Direction-Selective Inhibition in the Retina
Source: PLoS One. 2012 Apr 10;7(4):e35109. doi: 10.1371/journal.pone.0035109 (PMC3323634; doi:10.1371/journal.pone.0035109)

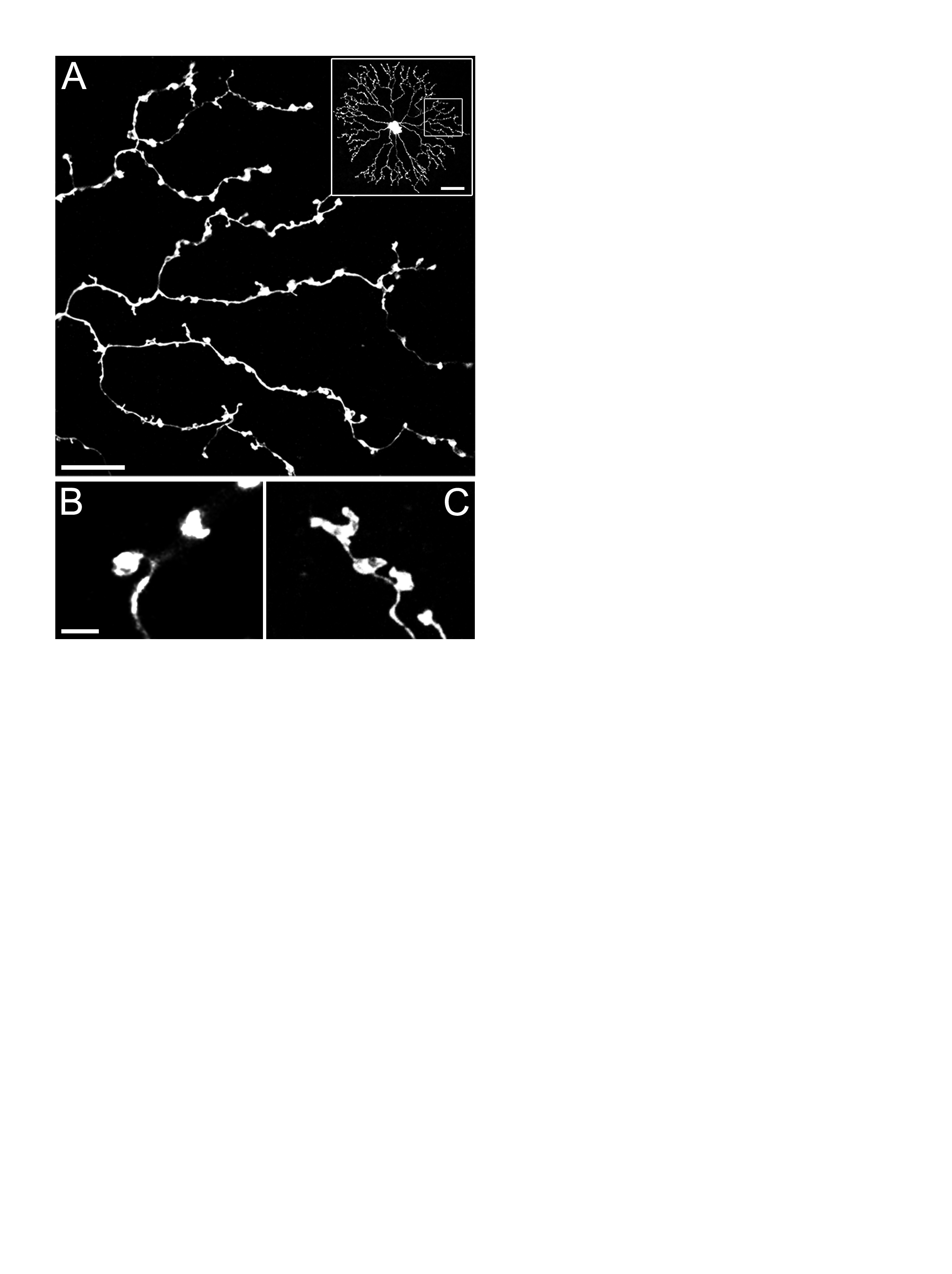

Supplement: Figure S1 — Morphology of varicosities on the distal dendrites of starburst amacrine cells (SACs). A. Distal dendrites of a dye-injected SAC (see inset) in a rabbit retina. B–C. High-magnification examples of varicosities, which are hook-like formations resulting from the presynaptic dendrite wrapping around the postsynaptic element [35], [47]. Scale bars: A, 15 µm (inset, 50 µm); B–C, 2 µm. (TIF) [file pone.0035109.s001.tif]

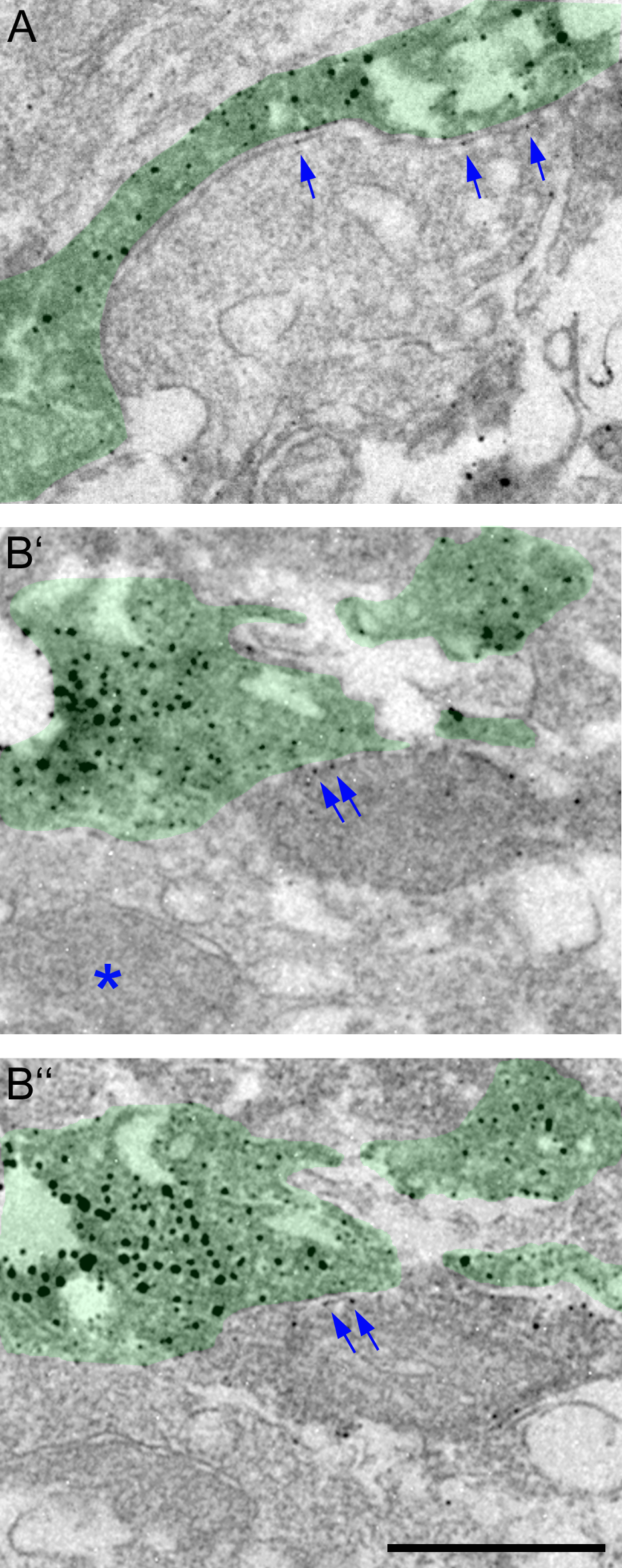

Supplement: Figure S2 — Electron micrographs of ultrathin sections of mouse retina, pre-embedded and double labeled for ChAT and GABAAR α2. Single section shown in A, two serial sections shown in B′–B″. The GABAAR α2 subunit is labeled with gold particles (tiny black dots indicated by arrows), which are visible on postsynaptic membranes apposed to ChAT-labeled SAC profiles, stained with DAB (big black dots, entire profiles overlaid in green). Note that the postsynaptic profiles do not contain vesicles, and are thus putative ganglion cell processes (compare to profile marked with asterisk in B′, which illustrates a typical presynaptic element, full of synaptic vesicles.) In B″ also note the elongated microtubule-like structures along the postsynaptic profile, typical for ganglion cell dendrites. Scale bar 0.5 µm. (TIF) [file pone.0035109.s002.tif]

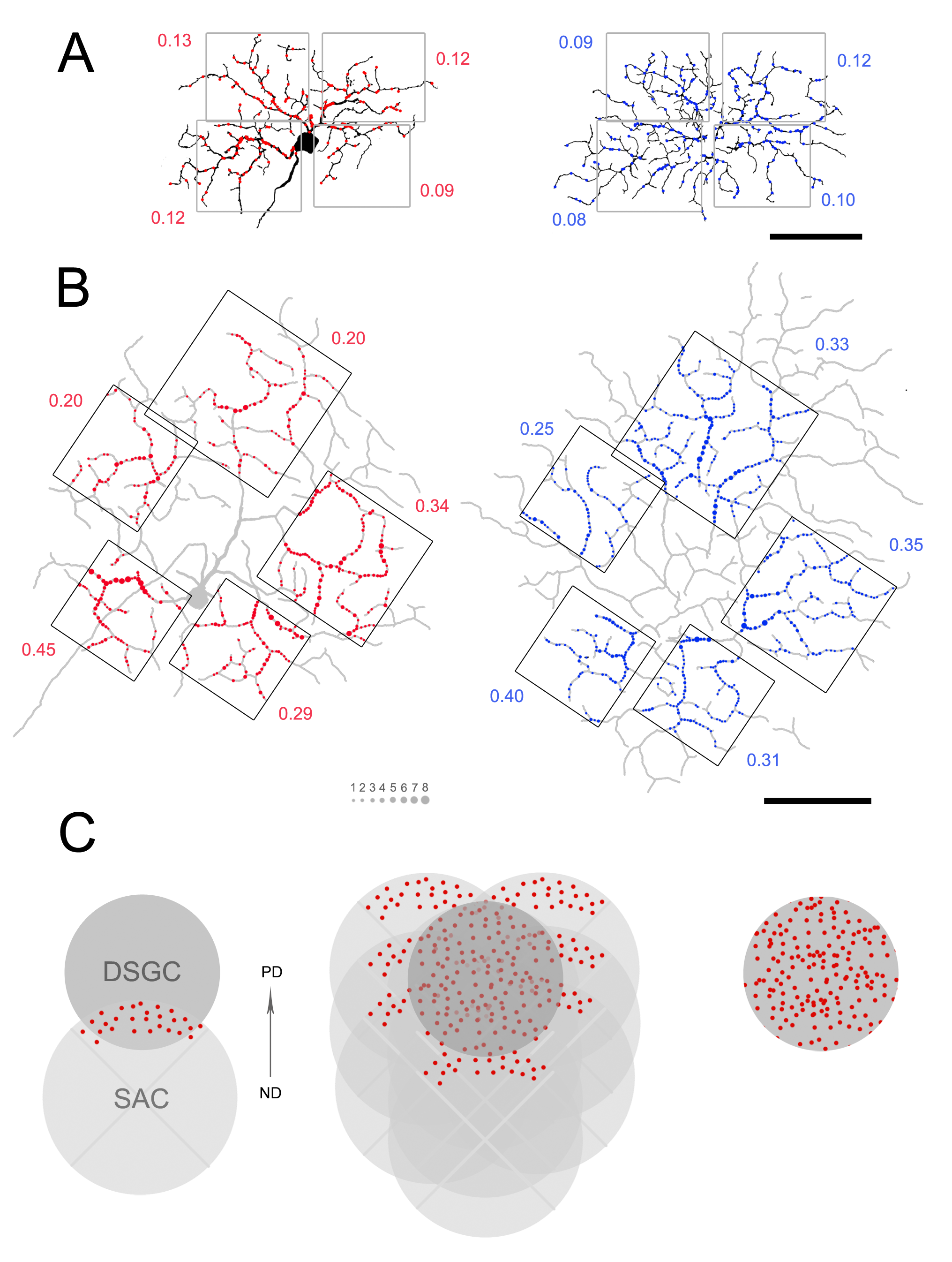

Supplement: Figure S3 — Spatial distribution of GABAA α2 receptor staining across the dendritic arbors of direction-selective ganglion cells (DSGCs). A–B. Reconstructions of putative ON-OFF DSGCs in mouse (A) and rabbit (B). The cell in the mouse retina was labeled in a transgenic GFP-O line, described in detail in [81]. ON and OFF dendritic trees are shown separately, with receptor puncta in red for the ON layer and blue for the OFF layer. In A, each dot represents a single receptor cluster (=an immunoreactive punctum); in B, dot size encodes number of receptor clusters (see scale beneath). Numbers indicate receptor cluster density per 100 µm dendritic length. The higher density of puncta found for the rabbit DSGC could reflect differences between species or, more likely, fixation degree. No obvious asymmetries can be seen across either cells or layers, as expected from recent statistical EM data [47] and illustrated in C. (While the values for the quadrants in B differ to some degree, regions with higher and lower densities are not segregated to particular sides of the dendritic field, e.g. null vs. preferred side.) C. SACs synapse onto DSGCs with different preferred directions (PD) with the constraint that each DSGC type is contacted by only those SAC dendrites oriented approximately along the DSGC's null direction (ND) (left). Together with the intrinsic DS tuning of the SAC output, this spatial arrangement warrants that SAC inhibition selectively vetoes the response to motion in null direction. Repeating this connectivity pattern for all SACs overlapping one given DSGC (middle) results in a relatively even distribution of receptors across the DSGC's dendritic tree (right). Scale bars: 100 µm. (TIF) [file pone.0035109.s003.tif]

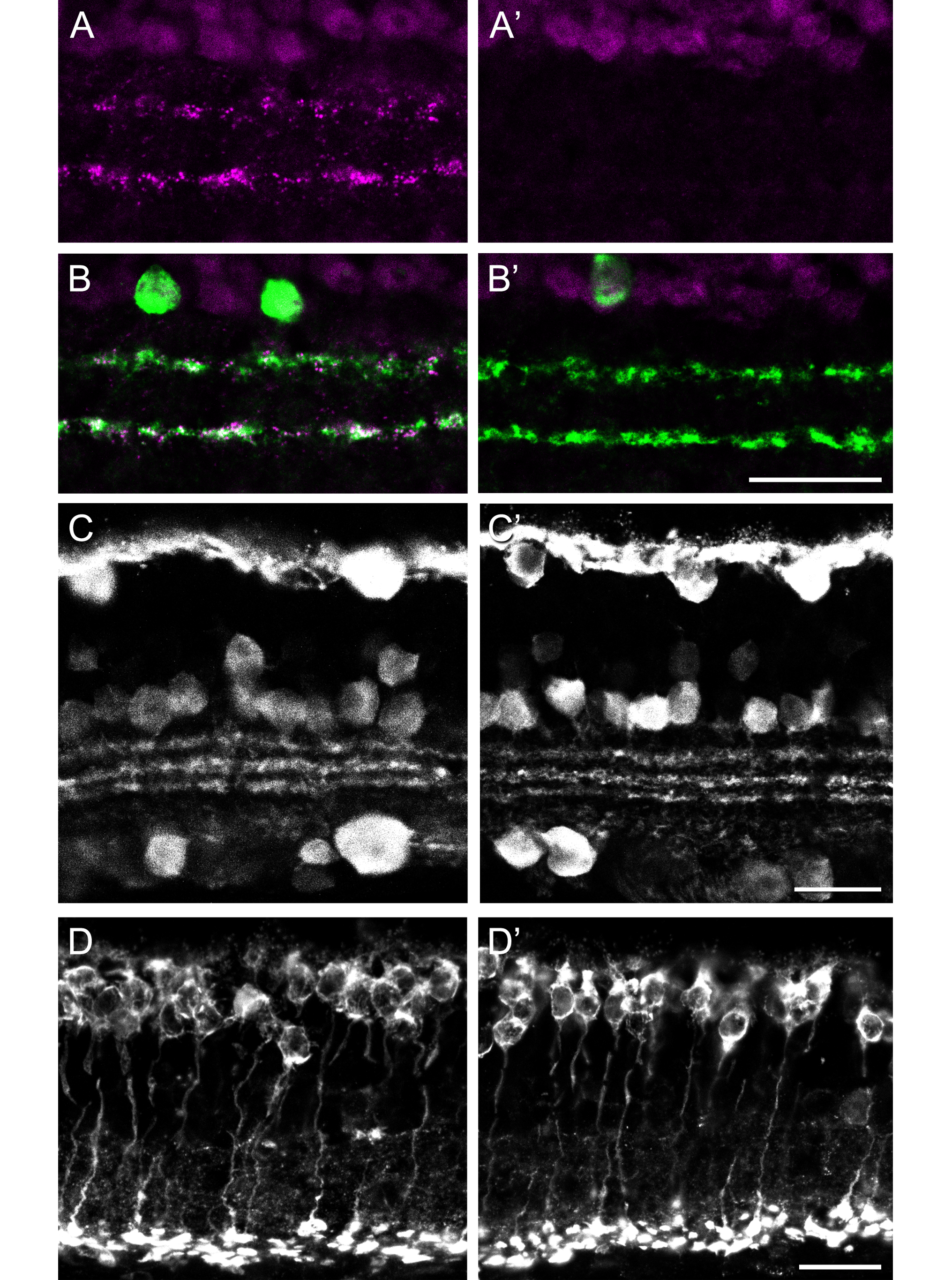

Supplement: Figure S4 — Retinal gross organization in GABAAR α2 knock-out mice. Vertical sections of wild-type (A–D) and GABAAR α2 KO retinae (A′–D′) immunolabeled against GABAAR α2 (magenta in A–A′ and B–B′), choline acetyl transferase (ChAT; green in B–B′), calbindin (C–C′), and protein kinase C alpha (D–D′). The retinae of the KO animals are void of α2 receptor staining, but otherwise do not show any obvious difference in gross retinal organization (e.g. thickness or lamination) when compared to the wild-type. Scale bars: 20 µm (B′ applies also to A, A′, B; C′ applies to C; D′ applies to D). (TIF) [file pone.0035109.s004.tif]
